# Involvement of Abscisic Acid in Transition of Pea (*Pisum sativum* L.) Seeds from Germination to Post-Germination Stages

**DOI:** 10.3390/plants13020206

**Published:** 2024-01-11

**Authors:** Galina Smolikova, Ekaterina Krylova, Ivan Petřík, Polina Vilis, Aleksander Vikhorev, Ksenia Strygina, Miroslav Strnad, Andrej Frolov, Elena Khlestkina, Sergei Medvedev

**Affiliations:** 1 Department of Plant Physiology and Biochemistry, St. Petersburg State University, 199034 St. Petersburg, Russia; e.krylova@vir.nw.ru (E.K.); s.medvedev@spbu.ru (S.M.); 2 Federal Research Center N.I. Vavilov All-Russian Institute of Plant Genetic Resources, 190000 St. Petersburg, Russia; khlest@bionet.nsc.ru; 3 Laboratory of Growth Regulators, The Czech Academy of Sciences, Institute of Experimental Botany & Palacky University, Faculty of Science, Slechtitelu 27, CZ-78371 Olomouc, Czech Republic; ivan.petrik@upol.cz (I.P.); miroslav.strnad@upol.cz (M.S.); 4 School of Advanced Engineering Studies, Novosibirsk State University, 630090 Novosibirsk, Russia; 5 Rusagro Group of Companies, 115054 Moscow, Russia; 6 Laboratory of Analytical Biochemistry and Biotechnology, K.A. Timiryazev Institute of Plant Physiology, Russian Academy of Sciences, 127276 Moscow, Russia; andrej.frolov@ipb-halle.de

**Keywords:** abscisic acid, DNA methylation, embryonic axis, ABA-associated genes, *Pisum sativum* L., seed-to-seedling transition

## Abstract

The transition from seed to seedling represents a critical developmental step in the life cycle of higher plants, dramatically affecting plant ontogenesis and stress tolerance. The release from dormancy to acquiring germination ability is defined by a balance of phytohormones, with the substantial contribution of abscisic acid (ABA), which inhibits germination. We studied the embryonic axis of *Pisum sativum* L. before and after radicle protrusion. Our previous work compared RNA sequencing-based transcriptomics in the embryonic axis isolated before and after radicle protrusion. The current study aims to analyze ABA-dependent gene regulation during the transition of the embryonic axis from the germination to post-germination stages. First, we determined the levels of abscisates (ABA, phaseic acid, dihydrophaseic acid, and neo-phaseic acid) using ultra-high-performance liquid chromatography–tandem mass spectrometry. Second, we made a detailed annotation of ABA-associated genes using RNA sequencing-based transcriptome profiling. Finally, we analyzed the DNA methylation patterns in the promoters of the *PsABI3*, *PsABI4*, and *PsABI5* genes. We showed that changes in the abscisate profile are characterized by the accumulation of ABA catabolites, and the ABA-related gene profile is accompanied by the upregulation of genes controlling seedling development and the downregulation of genes controlling water deprivation. The expression of *ABI3*, *ABI4*, and *ABI5*, which encode crucial transcription factors during late maturation, was downregulated by more than 20-fold, and their promoters exhibited high levels of methylation already at the late germination stage. Thus, although ABA remains important, other regulators seems to be involved in the transition from seed to seedling.

## 1. Introduction

In higher plants, seed production is crucial to species survival. Most seeds enter dormancy during late maturation and maintain this state until environmental conditions become favorable for germination [1,2]. The transition from dormancy to germination is influenced by a balance of phytohormones and significant environmental factors, such as temperature, water availability, and light [1,3]. This transition, occurring at the end of germination, involves extensive transcriptome reprogramming and signaling pathway alterations, leading to the silencing of seed maturation genes and activation of those for vegetative growth [4,5,6,7,8,9].

Whether seeds acquire the ability to germinate or remain dormant depends on the phytohormone balance [10,11,12]. Notably, abscisic acid (ABA) promotes seed dormancy and inhibits germination, while gibberellins (GAs) break seed dormancy and induce germination [13,14,15,16,17,18]. During early embryogenesis, ABA prevents seed abortion and promotes embryo growth, initially provided by the maternal tissues and later produced by the seeds themselves [18,19]. Consequently, the ABA level rises sharply late in embryogenesis, counteracting GAs and suppressing embryo growth [19].

As the embryo develops, it enlarges through cell elongation and accumulates storage compounds. ABA regulates the transport of monosaccharides and amino acids from maternal tissues and their conversion into stored forms like polysaccharides and proteins. In late maturation, metabolic processes slow down, and seeds desiccate and enter dormancy [20,21].

Numerous studies have shown that the decreasing ABA level is crucial for dormancy release and germination [7,12,19,22]. ABA degradation occurs through hydroxylation and conjugation, with ABA 8’-hydroxylases playing a key role in rapid ABA level decline during seed imbibition [17,23,24,25,26]. However, ABA’s signaling role during the seed-to-seedling transition remains unclear.

A key player in the seed transition from dormancy to germination is the LAFL regulatory network, comprising LEAFY COTYLEDON1 (LEC1) and LEC1-LIKE (L1L) of the NF-YB family transcription factors (TFs) and ABSCISIC ACID INSENSITIVE3 (ABI3), FUSCA3 (FUS3), and LEC2 (LEAFY COTYLEDON2) of the B3-AFL TF family [27,28,29]. The LAFL network, originating in a common ancestor of bryophytes and vascular plants, acts as a positive regulator of seed maturation genes but suppresses germination [30,31,32]. This network allows orthodox seeds to maintain desiccation tolerance during dormancy and germination [33,34,35,36]. Radicle protrusion marks the transition to the post-germination stage, with seeds becoming seedlings and losing desiccation tolerance [9,33,37]. This stage is typically associated with LAFL network silencing [5,32,34,38,39].

Our previous transcriptomic profiling of the *P. sativum* embryo axis before and after radicle protrusion revealed unexpected findings [4]. Although we anticipated the expression of LAFL network genes before radicle protrusion and their subsequent silencing, only *PsABI3* showed significant expression in the seed axis. We also observed the expression of other ABA-related genes (*PsABI4* and *PsABI5*). As result, *ABI3*, *ABI4*, and *ABI5* were expressed in the embryonic axis before radicle protrusion but downregulated at the post-germination stage. Given that *ABI3*, *ABI4*, and *ABI5* are central transcriptional factors in seed-specific events, including maturation, dormancy, longevity, germination, and post-germination growth [16,40,41], we propose that *PsABI3*, *PsABI4*, and *PsABI5* also play a role in regulating the *P. sativum* seed-to-seedling transition [4,9].

Germination-related repression of the LAFL transcriptional network is due to epigenetic regulation of gene expression through DNA methylation and post-translational modifications of histones [5,8,32,42,43,44]. DNA methylation patterns change throughout seed development, germination, and seedling establishment [8,45,46,47,48,49,50,51,52,53,54,55]. DNA methylation occurs in three sequence contexts (CG, CHG, and CHH) and refers to the addition of a methyl group to the C5 position of cytosine to form 5-methylcytosine [56]. Methylation of CHH sites notably increases from early to late stages of seed development, then decreases during germination [8,49,50]. Two DNA methylases, RdDM (RNA directed DNA methylation) and CMT2 (DOMAINS REARRANGED METHYLTRANSFERASE 2), responsible for methylating CHH sites in developing seeds, are inactivated during germination [53,54]. In contrast, CG and CHG methylation patterns are relatively stable throughout seed development [47,48,57]. Therefore, monitoring the level of 5-methylcytosine (m^5^C) is considered as a universal marker for seeds at the different stages of their ontogenesis [46].

This study analyzes ABA metabolite profiles, ABA-associated gene expression, and DNA methylation in the promoters of *PsABI3*, *PsABI4*, and *PsABI5* in the embryonic axis of germinated pea seeds before and after radicle protrusion. We discuss these findings in the context of ABA-dependent gene regulation during the seed-to-seedling transition.

## 2. Materials and Methods

### 2.1. Plant Material

Pea seeds of the commercial cultivar “Prima” were sourced from the N.I. Vavilov All-Russian Institute of Plant Genetic Resources, St. Petersburg, Russia. Seeds were imbibed for 72 h between layers of moist filter paper, then visually divided into two batches: (a) before embryonic root growth initiation (before radicle protrusion) and (b) post-initiation of root growth (after radicle protrusion). The seed axis from both batches was isolated, frozen in liquid nitrogen, homogenized, and stored at −80 °C before use in biochemical experiments and total genomic DNA extraction.

### 2.2. Quantitation of ABA and ABA-Related Metabolites

The selected plant hormones in the embryonic axis were quantified using ultra-high-performance liquid chromatography coupled with tandem mass spectrometry (UHPLC–MS/MS). The sample preparation and analysis were performed according to the modified protocol by Šimura and co-workers [58]. For the quantitation of ABA and ABA-related metabolites, 15 mg (fresh weight) of the homogenized plant material was extracted in 1 mL 60% (*v*/*v*) acetonitrile (ACN) with the addition of 5 pmol of [^2^H_6_]ABA as the internal standard. Four zirconium oxide 2.0 mm extraction beads (Next Advance, Troy, NY, USA) were added to the liquid sample. The sample was shaken in a Retsch MM400 bead mill (Retsch, Haan, Germany) at 27 Hz for 5 min, sonicated for 3 min, and incubated for half an hour at 4 °C. Afterwards, the sample was centrifuged at 20,000 rpm for 10 min at 4 °C (Allegra 64R benchtop centrifuge, Beckman Coulter, Brea, CA, USA). The supernatant was loaded onto an Oasis^®^ HLB 30 mg/L cc extraction cartridge (Waters, Milford, CT, USA). The cartridge was subsequently washed with 0.5 mL 60% (*v*/*v*) ACN and 0.5 mL 30% (*v*/*v*) ACN. All fractions (the flow-through and both washes) were collected and dried under reduced pressure using a SpeedVac concentrator (RC1010 Centrivap Jouan, ThermoFisher, Waltham, MA, USA). The sample was reconstructed in 40 µL of 25% (*v*/*v*) ACN and 5 µL of the sample was injected onto an Acquity UPLC CSH C18 RP 150 × 2.1 mm, 1.7 μm chromatographic column (Waters, Milford, CT, USA). The UHPLC separation was performed using the Acquity UPLC I-Class System (Waters, Milford, CT, USA) coupled to a triple quadrupole tandem mass spectrometer (Xevo TQ-XS) equipped with electrospray ionization (Waters, Manchester, UK). The gradient elution and the MS/MS working in multiple reaction monitoring (MRM) mode followed previously published conditions, as described by Šimura et al. [58]. The obtained chromatographic peaks were evaluated in MassLynx V4.2 software (Waters, Manchester, UK). The targeted compounds were quantified using the isotope dilution method.

### 2.3. Annotation of ABA-Associated Genes

ABA-associated genes were annotated based on RNA sequencing-based transcriptome profiling [4]. Annotation was performed utilizing the Ensembl BioMart tool (https://plants.ensembl.org/biomart/martview (accessed on 23 August 2023)) and the URGI database (https://urgi.versailles.inra.fr/Species/Pisum (accessed on 23 August 2023)). Gene ontology (GO) terms, InterPro domains (https://www.ebi.ac.uk/interpro (accessed on 23 August 2023)), and *Arabidopsis thaliana* orthologs were identified for each gene [59]. Genes with a false-discovery rate (FDR) <0.05 and log base 2-transformed fold change (|logFC|) >2 were considered differentially expressed. Clustering was performed using the k-means algorithm, and the optimal number of clusters was determined using the Elbow method.

### 2.4. DNA Extraction and Sodium Bisulfite Treatment

Total genomic DNA from seeds at two developmental stages (before and after radicle protrusion) was extracted using the DNeasy Plant Mini Kit (QIAGEN, Düsseldorf, Germany), according to the manufacturer’s instructions (www.qiagen.com (accessed on 10 March 2022)). Sodium bisulfite treatment of 1 μg genomic DNA from each sample was conducted using the EpiTect Fast Bisulfite Kit (QIAGEN, Germany).

### 2.5. Primer Design and In Silico Analysis

Primers for amplifying bisulfite-treated DNA were designed against cytosine-converted sequences using SnapGene 6.1.2 (https://www.snapgene.com (accessed on 21 February 2022)). Prediction of CpG islands in the *PsABI3*, *PsABI4*, and *PsABI5* promoter sequences was performed utilizing Meth-Primer 2.0 (https://www.urogene.org/methprimer2 (accessed on 21 February 2022)) and PlantPAN 3.0 (http://plantpan.itps.ncku.edu.tw/index.html (accessed on 25 February 2022)). Promoter mapping for transcription factor binding sites was performed using PlantPAN 3.0 and PCBase (http://pcbase.itps.ncku.edu.tw/index (accessed on 15 March 2022)), followed by filtering for stress and hormone response motifs at similar score = 1.

### 2.6. PCR, Electrophoretic Analysis, Extraction, and Purification

To amplify genomic and bisulfite-treated DNA, PCR was performed in a 50 μL mixture containing 70 ng of DNA template, 10 pM of each primer, and BioMaster HS-Taq PCR kit (2×) (BioLabMix, Novosibirsk, Russia) or Tersus Plus PCR kit (Evrogen, Moscow, Russia), according to the manufacturer’s instructions. The PCR conditions included an initial denaturation step at 94 °C for 5 min; followed by 35 cycles of denaturation at 94 °C for 1 min, annealing 50 °C for 1 min, and extension at 72 °C for 2 min; and a final elongation step at 72 °C for 5 min. The PCR screening of colonies was performed in a 25 μL mixture containing 10 pM of M13F and M13R primers (Evrogen, Russia), 0.25 mM of each dNTP, 1× reaction buffer (67 mM TrisHCl, pH 8.8; 2 mM MgCl_2_; 18 mM (NH_4_)_2_SO_4_; 0.01% Tween 20) and 0.5 U Taq polymerase (Syntol, Russia). After an initial denaturation at 95 °C for 15 min; 35 cycles were performed at 94 °C for 20 s, 55 °C for 30 s, and 72 °C for 1 min; followed by a final elongation at 72 °C for 5 min. Electrophoretic analysis was performed on 1% agarose gel (Helicon, Moscow, Russia) prepared on TAE buffer (Sigma-Aldrich, St., Louis, MO, USA) with ethidium bromide (VWR (Amresco), Cleveland, OH, USA). The amplified fragments were extracted from the gel using the MinElute Gel Extraction Kit (QIAGEN, Germany).

### 2.7. Cloning and Sequencing of the Amplified PCR Fragments

Freshly prepared PCR products were ligated with a vector using the Quick-TA kit (Evrogen, Russia), which included the pAL2-T vector, Quick-TA T4 DNA Ligase, buffer, M13 forward primer, and M13 reverse primer, according to the manufacturer’s instructions. Chemical transformation of competent *Escherichia coli* (Migula 1895) Castellani and Chalmers 1919 DH10B cells was then performed. Transformed colonies carrying inserts of the expected size were selected on selective LB medium (DIA-M, Moscow, Russia) with 100 µg/mL of ampicillin (BioChemica, PanReac Applichem, Spain). The purified amplified fragments were sequenced in both directions using M13 primers and the BigDye™ Terminator v3.1 Cycle Sequencing Kit (Applied Biosystems™, Waltham, MA, USA) on a 3500 Applied Biosystems Genetic Analyzer. For DNA methylation analysis, at least 10 clones were sequenced for each amplicon. The alignment of sequences was carried out using SnapGene 6.1.2 (https://www.snapgene.com/ (accessed on 10 December 2022)).

### 2.8. Statistical Analyses

Two-tailed *t*-tests (alpha = 0.05) were used to compare the means of ABA-related metabolites. Analysis was performed using MS Excel add-in, with data representing the mean ± standard error of 3 biological and 3 technical replicates (*n* = 9).

## 3. Results

### 3.1. Quantitation of ABA and ABA-Related Metabolites in the Pea Embryonic Axis before and after Radicle Protrusion

**Figure 1:**
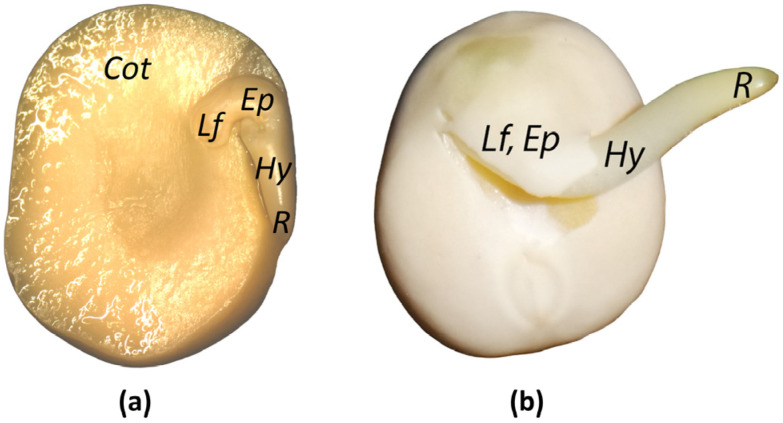
Images of *P. sativum* seeds: (**a**) before radicle protrusion, (**b**) after radicle protrusion. Embryo includes cotyledons (*Cot*), first true leaves (*Lf*), epicotyl (*Ep*), hypocotyl (*Hy*), and root (*R*). Embryonic axis includes *Lf*, *Ep*, *Hy*, and *R*.

To delve deeper into ABA homeostasis, we examined the levels of ABA and its metabolites in the embryonic axis of germinated pea seeds, both before and after radicle protrusion. This axis encompasses the first true leaves, epicotyl, hypocotyl, and root (Figure 1).

**Figure 2:**
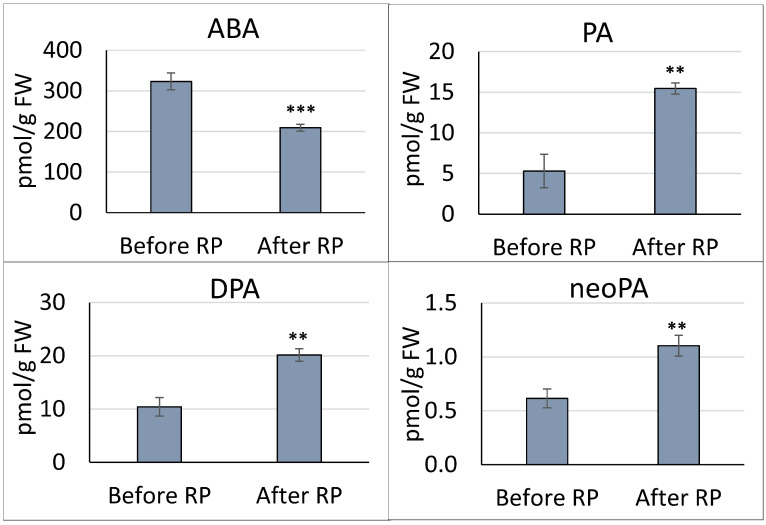
The contents of abscisic acid (ABA), phaseic acid (PA), dihydrophaseic acid (DPA), and neo-phaseic acid (neoPA) observed in the embryonic axis of *P. sativum* before and after radicle protrusion (RP). The data represent the mean ± standard error of 9 biological replicates. The statistical analysis relied on two-tailed *t*-test with a critical alpha value of 0.05. Significant differences between the mean values are indicated (*** *p* ≤ 0.001, ** *p* ≤ 0.005).

We analyzed levels of abscisic acid (ABA), phaseic acid (PA), dihydrophaseic acid (DPA), neo-phaseic acid (neoPA), and 7′-hydroxy ABA (7′-OH-ABA). Notably, we observed an accumulation of PA and DPA, which are key products of ABA catabolism, against a backdrop of decreasing ABA level (Figure 2). Intriguingly, 7′-OH-ABA was not detected in the embryonic axis before or after radicle protrusion.

### 3.2. Categorization and Functional Annotation of ABA-Associated DEGs in the Pea Embryonic Axis before and after Radicle Protrusion

In our previous work, we performed RNA sequencing of the isolated embryonic axis before and after radicle protrusion [4]. Here, we provide a more detailed profile of ABA-associated differentially expressed genes (DEGs) annotated using the Pea Genome Assembly *v1a* from the UGRI server as the primary annotation source [60]. The differentially expressed genes were annotated using a BLASTX search against the *A. thaliana* (TAIR 10) protein database (with a threshold *e*-value < 10^−9^). GO and MapMan annotations were assigned based on *A. thaliana* homologous proteins. Singular enrichment analysis of the DEG lists was performed using the AgriGO v.2 toolkit [61]. GO terms with adjusted *p*-value < 0.05 were considered significantly enriched. We found 30 *A. thaliana* genes belonging to the GO term «Response to abscisic acid stimulus» and 70 orthologs in *P. sativum*.

**Figure 3:**
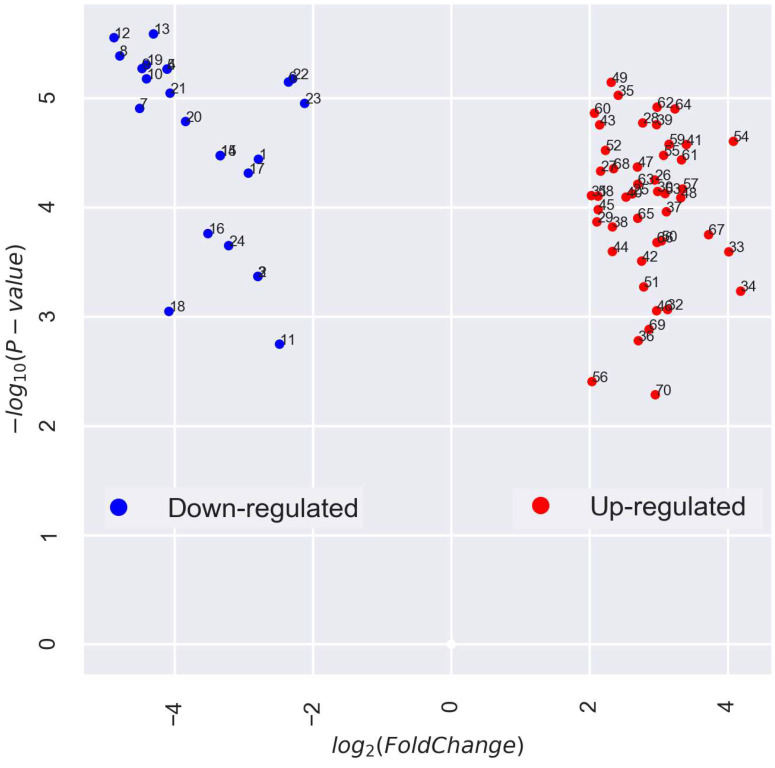
Volcano plot representing 70 differentially expressed genes (DEGs). The X-axis indicates the log2-transformed gene expression fold changes in the seed axis before and after radicle protrusion. The Y-axis indicates the log10-transformed *p*-values. Significant DEGs with lower expression are highlighted in blue (№ 1–24). Significant DEGs with higher expression are highlighted in red (№ 25–70). See Table S1 for the full description of the downregulated and upregulated genes.

Thus, a total of 70 ABA-associated DEGs were annotated in the pea embryonic axis. Among these, 46 genes showed higher expression and 24 genes showed lower expression after radicle protrusion by more than 4-fold (|logFC| > 2) (Figure 3 and Figure S1, Table S1).

ABA-dependent DEGs upregulated in the seed axis after radicle protrusion included those related to cellular signaling, stress resistance, membrane transporters, and TFs regulating developmental programs (Table S1). These genes encoded serine-threonine/tyrosine protein kinases CRK29 (*Psat6g212040*) and CIPK17 (*Psat0s2012g0280*), a protein phosphatase 2C family member (*Psat7g017080*), the *α*-subunit of G protein (*Psat6g097080*), and an inositol polyphosphate-related phosphatase (*Psat4g078320*). The expression of genes associated with the water deprivation response, antifungal proteins, and calcium signaling also significantly increased. The expression of *Psat6g199400* encoding protein RD29B/LTI65 increased 4.5-fold, that of *Psat5g266320* encoding antifungal protein ginkbilobin-2 increased 5–8-fold, and that of *Psat4g146960* encoding calcium signaling protein ANNEXIN4 increased 9-fold. Genes responsible for the synthesis of membrane transporters included *Psat4g117800* (encoding P-ATPase) and *Psat4g184760* (encoding potassium channel AKT2/3). The expression of *Psat2g121520* (encoding TCP15 protein) increased 8-fold.

Conversely, the downregulated DEGs included key ABA-response genes like *ABI5* (*Psat3g033680*), *ABI3* (*Psat3g142040*), *ABI4* (*Psat2g031240*), *LTI65* (*Psat0s2227g0040*), *LTP4* (*Psat7g227120*), *HVA22E* (*Psat5g052360*), and *RD22* (*Psat6g033920* and *Psat6g033960*) (Table S1). These genes are highly conserved across functional domains, with *ABI4*, *ABI5*, and *HVA22E* exhibiting sequence homology in various drought-tolerant species [4]. These genes may play a crucial role in dehydration tolerance during the transition from seed germination to seedling establishment.

### 3.3. DNA Methylation in the Promoters of the PsABI3, PsABI4, and PsABI5 Genes

We selected the *PsABI3* gene along with newly identified drought-responsive genes *PsABI4* and *PsABI5* for epigenetic analysis. These genes were identified in the *P. sativum* genome and sequenced from the commercial cultivar “Prima” (Table S2).

In silico analysis of the promoters and first exons (including 5′-UTR) of *PsABI3*, *PsABI4*, and *PsABI5* revealed low GC composition (29%, 34%, and 23% respectively), with only individual CpG sites predicted and no CpG islands detected (Figure S2). Considering that plant DNA methylation can occur at CpG, CpHpG, and CpHpH sites, we designed primers for bisulfite sequencing (with conversion of unmethylated C to T) of both CpG and non-CpG sites (Table S2).

**Figure 4:**
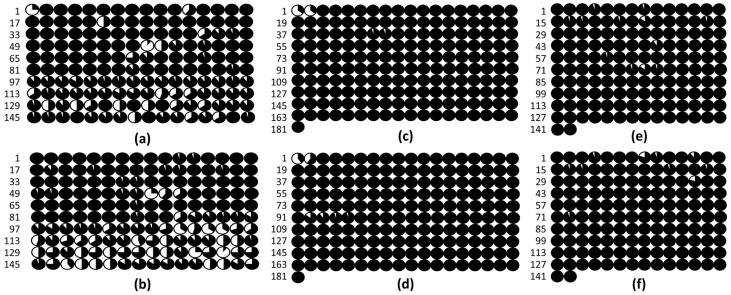
Methylation of ABA*-*related gene promoters in the embryonic axis of germinated *P. sativum* seeds before and after radicle protrusion (RP). (**a**) The *PsABI3* gene promoter before RP. (**b**) The *PsABI3* gene promoter after RP. (**c**) The *PsABI4* gene promoter before RP. (**d**) The *PsABI4* gene promoter after RP. (**e**) The *PsABI5* gene promoter before RP. (**f**) The *PsABI5* gene promoter after RP. The length of the analyzed segment of the *PsABI3* promoter is 1057 bp and the number of cytosines is 160; for the PsABI4 promoter, the length is 721 bp with 181 cytosines; and for the PsABI5 promoter, the length is 1231 bp with 142 cytosines. Circles represent cytosines, with methylated bases shown in black and unmethylated bases in white. See Figures S3–S5 for mapping of *PsABI3*, *PsABI4,* and *PsABI5*, accordingly.

To analyze the methylation profile of the promoters and the beginning of the first exons of the *PsABI3*, *PsABI4* and *PsABI5* genes, we performed amplification of the bisulfite-treated DNA using designed primers (Table S3). Bisulfite-treated DNA amplification and subsequent cloning revealed methylation in the promoters of *PsABI3*, *PsABI4*, and *PsABI5* already before radicle protrusion (Figure 4).

Additionally, we mapped the promoters of these genes to compare potential methylation sites and binding sites for TFs (Table S4). Notably, the *PsABI4* promoter had the lowest number of TF binding sites, while the *PsABI5* promoter contained numerous potential LAFL protein binding sites, along with motifs associated with responses to cold and water deprivation.

## 4. Discussion

### 4.1. ABA Catabolism

**Figure 5:**
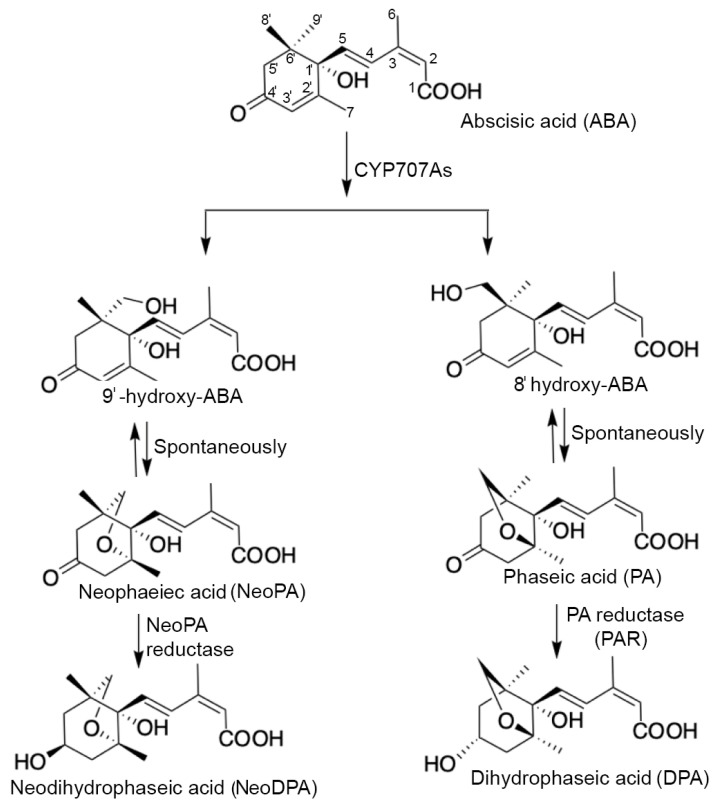
Oxidative pathways of ABA catabolism: CYP707s—cytochrome P450 monooxygenases; PA—phaseic acid; neoPA—neophaseic acid; DPA—dihydrophaseic acid; neoDPA—epi-neodihydrophaseic acid; PAR—PA reductase.

ABA plays vital roles in seed development and maturation, encompassing the accumulation of storage compounds, acquisition of desiccation tolerance, induction of dormancy, and suppression of precocious germination [12,17,18,19,62,63,64]. However, to break dormancy and initiate germination, ABA needs to be catabolized, primarily through hydroxylation and conjugation. The primary ABA hydroxylation route is the ABA catabolic pathway (Figure 5), which relies on the activities of CYP707A cytochrome P450, notably ABA 8′-hydroxylases [65].

Initially, ABA is catalyzed by 8′-hydroxylase, converting it to 8′-hydroxy ABA (8′-OH ABA), an unstable intermediate [66,67]. This intermediate is then spontaneously rearranged into PA and subsequently reduced by PA reductase (PAR) to DPA [24,68]. The 9′-hydroxylation pathway, similar to 8′-hydroxylation, involves CYP707A enzymes and converts 9′-hydroxy ABA (9′-OH ABA) to neoPA with both 8′-C and 9′-C hydroxylation catalyzed by the same enzyme [16]. Recently, Bai et al. (2022) [24] identified a downstream catabolite of neoPA in the 9′-hydroxylation pathway as epi-neodihydrophaseic acid (*epi*-neoDPA) and discovered the responsible enzyme, neoPA reductase 1 (NeoPAR1) (Figure 5).

Our study examined ABA and ABA-related catabolites in the embryonic axis of *P. sativum* seeds before and after radicle protrusion. We found a decline in ABA content with a concurrent rise in levels of its catabolites (PA, DPA, and neoPA) (Figure 2). Intriguingly, PA, similar to ABA, can regulate stomatal closure and suppress seed germination [69,70]. Weng et al. (2016) demonstrated that PA functions as a signaling molecule through ABA receptors. Similar ABA-like hormonal activity was observed for neoPA, but not for *epi*-neoDPA [24]. Additionally, altered seed germination patterns were noted in neo-PAR1 mutant and overexpression lines, implicating the ABA catabolic pathway as a critical regulatory mechanism during the seed-to-seedling transition [24]. Despite the reduced ABA level, the accumulation of its catabolic products (PA and neoDPA) in the embryonic axis suggests a continued regulatory influence via ABA receptors.

### 4.2. Annotation of ABA-Associated DEGs

In our prior RNA sequencing-based transcriptomic analysis of the pea embryonic axis isolated from seeds before and after radicle protrusion [4], we identified 24,184 DEGs, with 2101 showing notably higher expression. This work extends that analysis by focusing on ABA-associated DEGs (ABA-DEGs). Of the 70 ABA-DEGs annotated, 46 genes were upregulated and 24 genes were downregulated by more than 4-fold after radicle protrusion (Figure 3).

The upregulated ABA-DEGs predominantly pertained to cellular signaling, stress resistance, membrane transporters, and transcription factors that regulate seedling development. For instance, *Psat6g199400*, encoding RD29B/LTI65, which responds to water deprivation, was upregulated 4.5-fold. This gene’s promoter region contains two ABA-responsive elements (ABREs) that require *cis*-acting elements for the dehydration-responsive expression of RD29B/LTI65 [71,72]. Similarly, *Psat4g146960*, encoding ANNEXIN4, a calcium-binding protein involved in drought and other stress responses [73,74], showed a 9-fold increase in expression (Table S1).

Among the upregulated genes were those coding for membrane transporters like *Psat4g117800* (P-ATPase) and *Psat4g184760* (potassium channel AKT2/3). P-type ATPases play a role in ion transport across membranes, utilizing ATP for transmembrane conformational changes [75,76]. Additionally, *Psat2g121520*, encoding TCP15, a transcription factor implicated in cell expansion and proliferation [77,78], was upregulated 8-fold. The TCP proteins, known as TEOSINTE BRANCHED 1 (TB1) in maize, CYCLOIDEA (CYC) in *Anthirrinum majus*, and PCF in rice [79], have been linked to various developmental processes, including light-induced cotyledon opening in *Arabidopsis* [80].

Conversely, the downregulated ABA-DEGs included genes central to ABA signaling (*ABI3*, *ABI4*, and *ABI5*) and those involved in the water deprivation response (*LEA14*, *RD22*, *HVA22*, *PER1*, and *LTI65*) (Table S1). Seed germination is governed by the antagonistic balance of ABA/GA, with ABA catabolism preceding GA synthesis and activation [5,7,17]. Key ABA signaling genes *ABI3*, *ABI4*, and *ABI5* encode the TFs featuring B3, AP2, and bZIP domains, which control the expression of ABA-responsive genes crucial for seed maturation, dormancy, longevity, germination, and post-germination growth ([12,16,81,82,83].

*ABI5* encodes a member of the basic leucine zipper TF family and is involved in ABA signaling in seeds by acting as a signal integrator between ABA and other hormones [41,84,85]. Arabidopsis *abi5* mutants have pleiotropic defects in the ABA response, including reduced sensitivity to ABA, inhibition of germination, and altered expression of some ABA-regulated genes [86,87]. Notably, *Psat3g033680*, encoding ABI5, exhibited 22-fold downregulation after radicle protrusion.

ABI4 was shown to be a key integration node for multiple signals participating in critical transition steps during plant ontogenesis [88,89,90]. In dormant seeds, ABI4 acts as a repressor of ABA catabolism by binding to the promoter of CYP707A, being the main enzyme of ABA catabolism [91]. Thereby, ABA and GAs can antagonistically modify the expression and stability of *ABI4*, suggesting the existence of regulatory loops [88]. In germinating seeds, ABI4 can regulate both ABA synthesis and catabolism. Some authors suggest that ABI4 is a key regulator of the balance between ABA and GAs in seeds at the post-germination stage [88,90]. In our study, the level of the *Psat2g031240* gene encoding ABI4 was decreased 21-fold.

*ABI3* encodes AP2/B3-like transcriptional factor family protein [92]. ABI3 belongs to the LAFL regulatory network, where it interacts with LEAFY COTYLEDON1 (LEC1), ABSCISIC ACID INSENSITIVE3 (ABI3), FUSCA3 (FUS3), and LEC2 [31,32]. The LAFL network is a positive regulator of seed dormancy and needs to be suppressed for seed germination. Together, ABI3, FUS3, and LEC1 are involved in the sensitivity of seeds to ABA and regulate expression of the 12S storage protein gene family [93]. In addition, both FUS3 and LEC1 positively regulate ABI3 protein abundance in seeds [94]. The expression of *Psat3g142040*, encoding ABI3, was decreased 21-fold.

We also found the downregulation of ABA-dependent genes involved in the response to water deprivation (*LEA14*, *RD22*, *HVA22*, *PER1*, and *LTI65*) (Table S1). In accordance with our findings, *Psat7g085840* encoding peroxiredoxin1 (PER1), *Psat0s2227g0040* encoding protein LTI65/78, and *Psat0s2780g0040* encoding late embryogenesis abundant (LEA) protein were downregulated 20–30-fold. Peroxiredoxins are thiol-dependent antioxidants containing one (1-Cys) or two (2-Cys) conserved Cys residues [95]. *PER1* encodes a 1-Cys peroxiredoxin (PER1) protein that accumulates during seed development but rapidly disappears upon germination [96]. PER1 is involved in the quenching of reactive oxygen species (ROS) during late maturation, dormancy, and early germination, thereby maintaining seed viability [96,97,98]. The low temperature-induced (LTI) protein family is associated with responses to abiotic stresses. In Arabidopsis, homologous genes *RD29A (LTI78)* and *RD29B (LTI65)* are induced by cold, drought, salt, and abscisic acid [71]. Most LEA genes have ABA response elements in their promoters and their expression can be induced not only by ABA, but also by cold or drought. Desiccation-related protein LEA14 belongs to the group II LEA proteins, also known as dehydrins [99]. LEA14 is induced in response to salt and low temperature [100].

### 4.3. Epigenetic Regulation of the PsABI3, PsABI4, and PsABI5 Genes Based on DNA Promoter Methylation

Major transitions in the plant life cycle require fine-tuned regulation at the molecular and cellular levels. Epigenetic regulation, particularly DNA methylation, is crucial for maintaining genome stability in plants by inhibiting transposable element movement and modulating gene expression during development and stress responses [8,56]. DNA methylation patterns in seeds undergo significant changes during their development and germination [8,46,47,48,49,52,54].

Our study reveals that during the transition from germination to post-germination, the expression of key ABA signaling pathway genes (*ABI3*, *ABI4*, and *ABI5*) is markedly suppressed. We analyzed the DNA promoter methylation profiles of *PsABI3*, *PsABI4*, and *PsABI5* to understand their epigenetic regulation. Contrary to our expectations of low promoter methylation levels based on their expression before radicle protrusion [4], we observed high methylation levels both before and after this developmental stage (Figure 4). Notably, approximately one-third of the *PsABI3* gene promoter region showed reduced methylation. However, this region might belong to the 5′-UTR as per the Pea Genome International Consortium version 1a (Figure S3, pink).

We further investigated the coincidence of epigenetic markers with transcription factor binding sites in the promoters of these genes using PlantPAN 3.0 and PCBase, focusing on stress and hormone response motifs. *PsABI5* showed numerous potential binding sites for LAFL network proteins, along with motifs associated with cold and water deprivation responses (Figure S4). This finding aligns with the role of ABI5 as a major regulator of seed maturation and longevity in legumes [41]. Our results suggest that epigenetic modifications impacting the binding ability of *ABI3*, *ABI4*, and *ABI5* to DNA promoters occur prior to initiation of the seed transition from germination to post-germination.

Thus, our study provides insight into the involvement of ABA in the transition of *P. sativum* from the germination to post-germination stages when seeds turn into seedlings. The initiation of embryonic axis growth corresponds with changes in the abscisate profile: a decrease in the ABA level and an accumulation of its catabolites (PA, DPA, and neoPA), which possess hormonal activity similar to ABA [24,101]. Our in-depth analysis of ABA-DEGs revealed 46 upregulated and 24 downregulated genes with more than 4-fold changes. Most upregulated ABA-DEGs were related to the regulation of seedling development. Most notably, the expression of *ABI3*, *ABI4*, and *ABI5* was significantly downregulated, and their promoters exhibited high levels of methylation both before and after radicle protrusion. While ABA continues to be important, other regulators appear to be involved in the seed-to-seedling transition.

## Data Availability

Data are contained within the article and supplementary materials.

## Supplementary Materials

The following supporting information can be downloaded at: https://www.mdpi.com/article/10.3390/plants13020206/s1, Figure S1: Expression heatmap of ABA-associated DEGs in the embryonic axis of *P. sativum* before and after radicle protrusion (RP); Figure S2: Predicted methylation sites for CpG motifs in the sequences of seed resistance to dehydration genes in the *P. sativum* genome; Figure S3: Mapping of the *PsABI3* gene promoter; Figure S4: Mapping of the *PsABI4* gene promoter; Figure S5: Mapping of the *PsABI5* gene promoter; Table S1: Annotation of ABA-associated genes in the transcriptome of the *P. sativum* embryonic axis; Table S2: The ABA-dependent genes of *P. sativum* seeds selected for analysis of DNA methylation in the promoters; Table S3: Primers used for amplification of gene promoter regions from bisulfite-treated DNA isolated from the embryonic axis of *P. sativum*; Table S4: Functions of transcription factors, the binding sites of which were identified in the gene promoter regions of *PsABI3*, *PsABI4,* and *PsABI5* [102,103,104,105].
