# Supplementary material for: Involvement of Abscisic Acid in Transition of Pea (Pisum sativum L.) Seeds from Germination to Post-Germination Stages
Source: Plants (Basel). 2024 Jan 11;13(2):206. doi: 10.3390/plants13020206 (PMC10819913; doi:10.3390/plants13020206)
Supplement: Supplementary file 1 [file plants-13-00206-s001.zip › Supplementary_Figures S1-S5.pdf]

Supplementary Figures

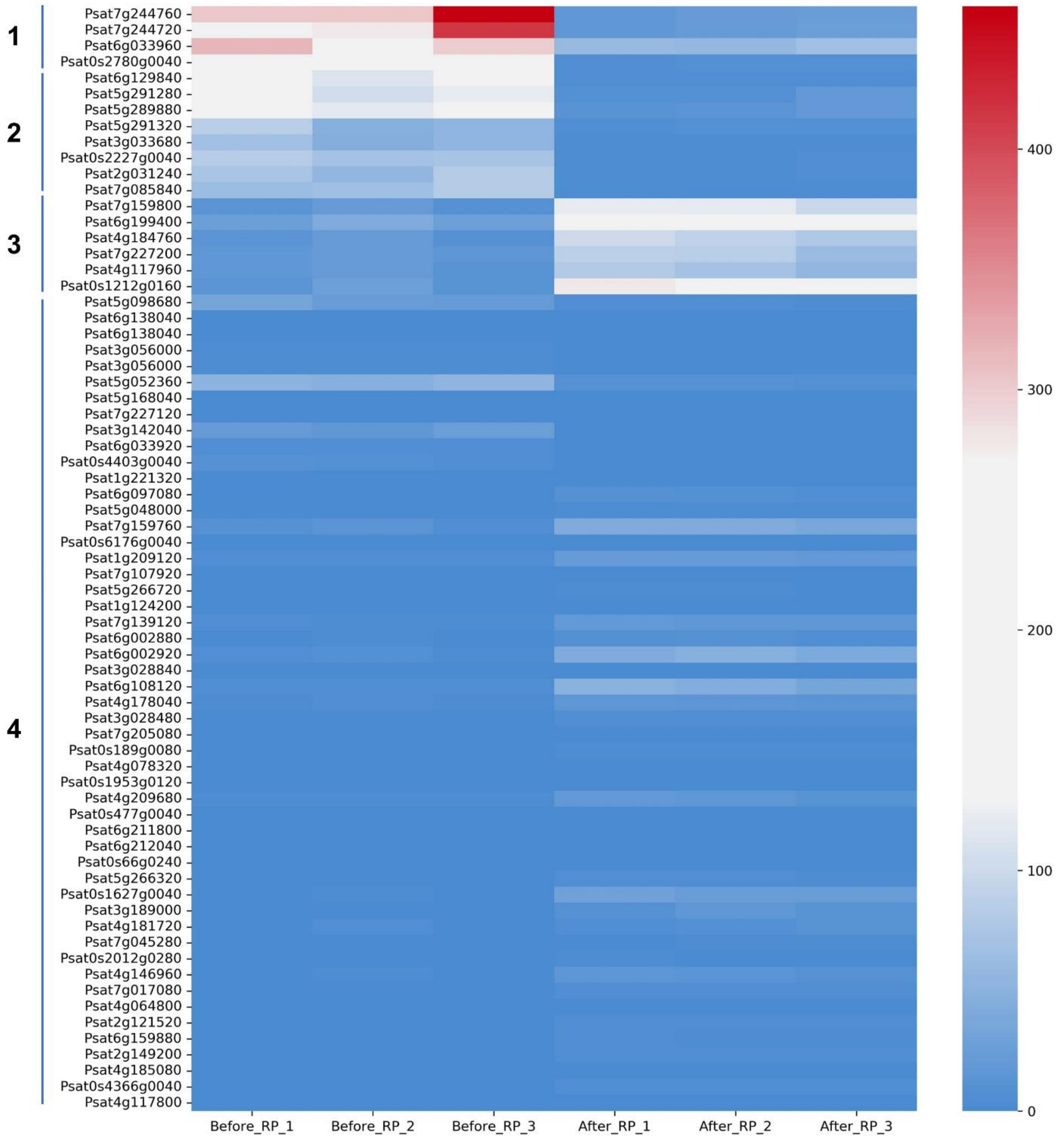

Figure S1. Expression heatmap of ABA-associated DEGs in embryonic axes of P. sativum before and after radicle protrusion (RP) The red and blue color denotes high and low values of RPKM-normalized gene expression, respectably. The red-white-blue gradient represents the change of values from high to low. 1-4 – clusters of genes with similar expression behavior (obtained using k-means algorithm.)

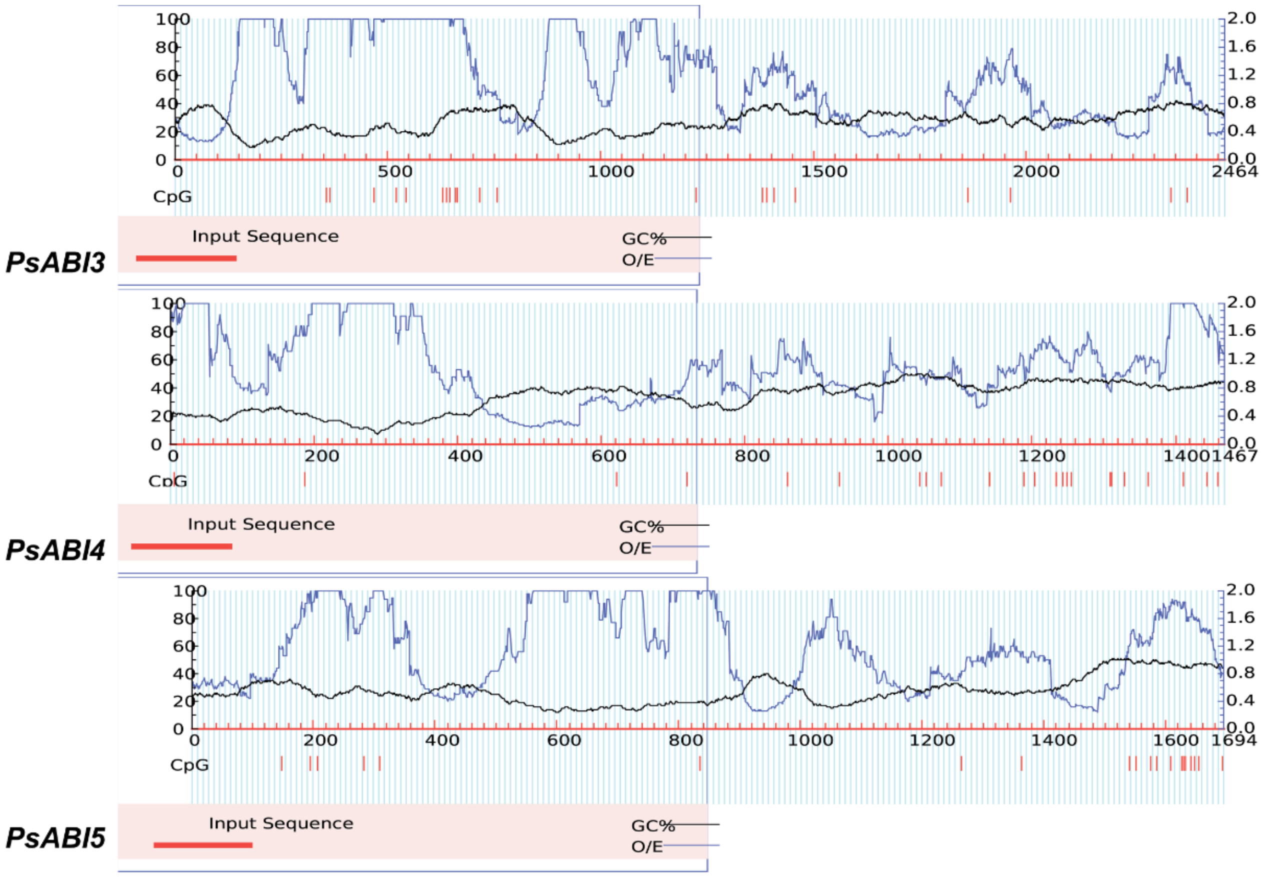

Figure S2. Predicted methylation sites for CpG motifs in the sequences of the seed resistance to dehydration genes in the *P. sativum* genome. CpG sites are marked with red vertical lines. GC% – black line. O / E – blue line.

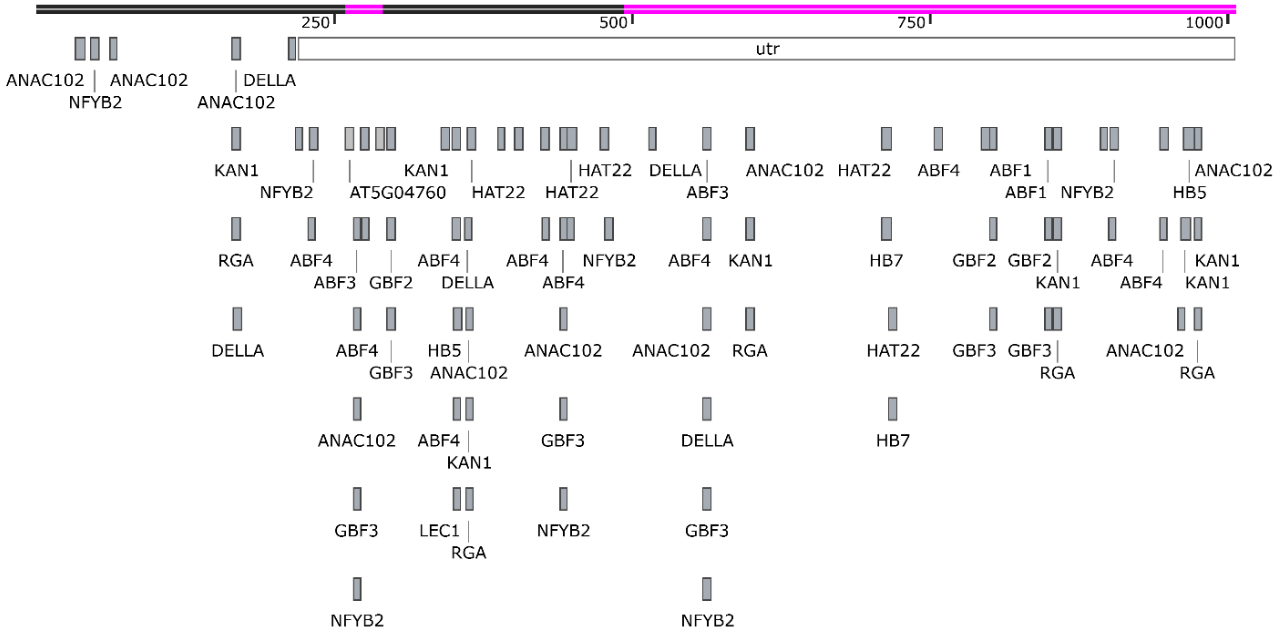

Figure S3. Mapping of the Ps*ABI3* gene promoter.

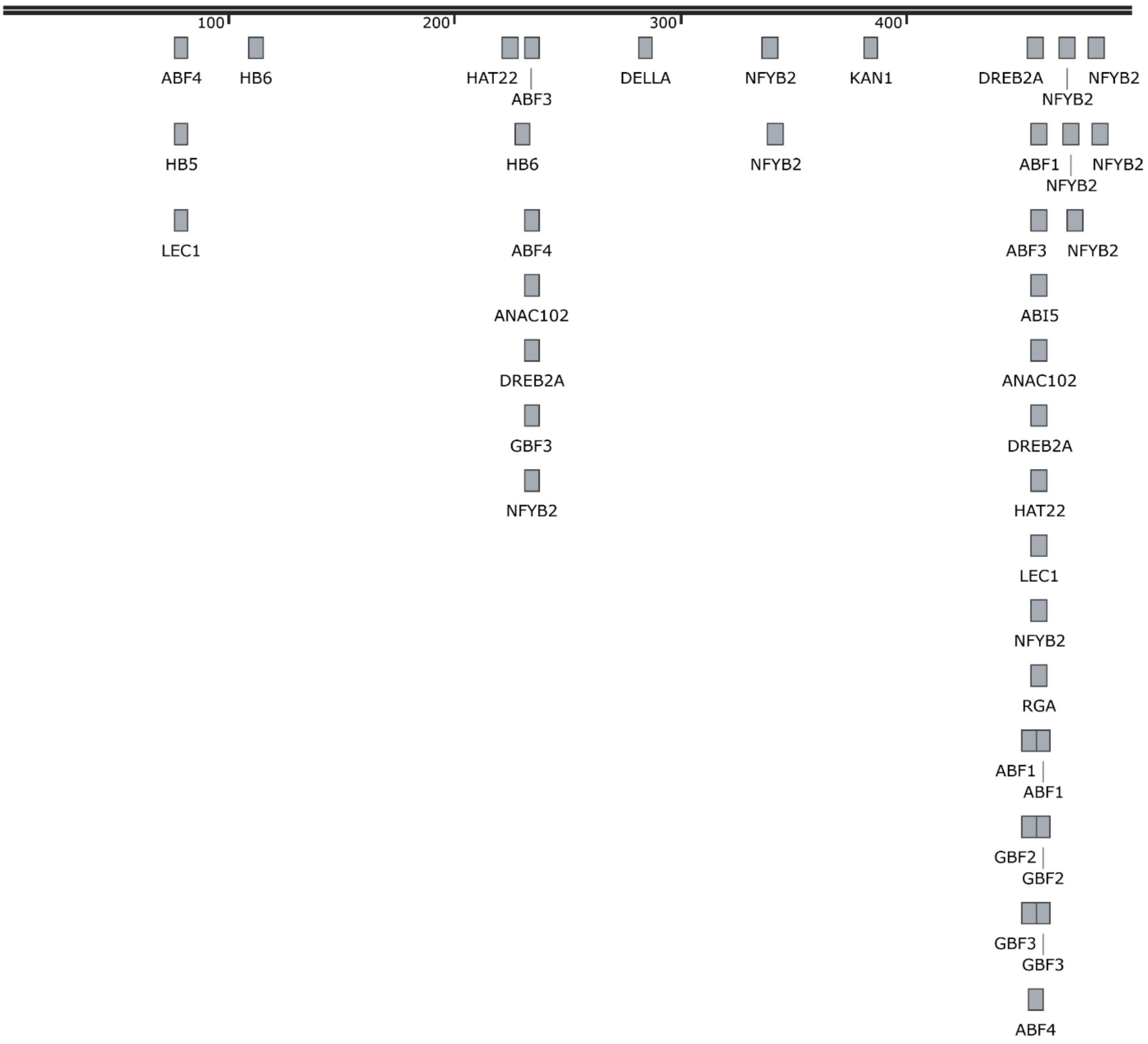

Figure S4. Mapping of the *PsABI4* gene promoter**.**

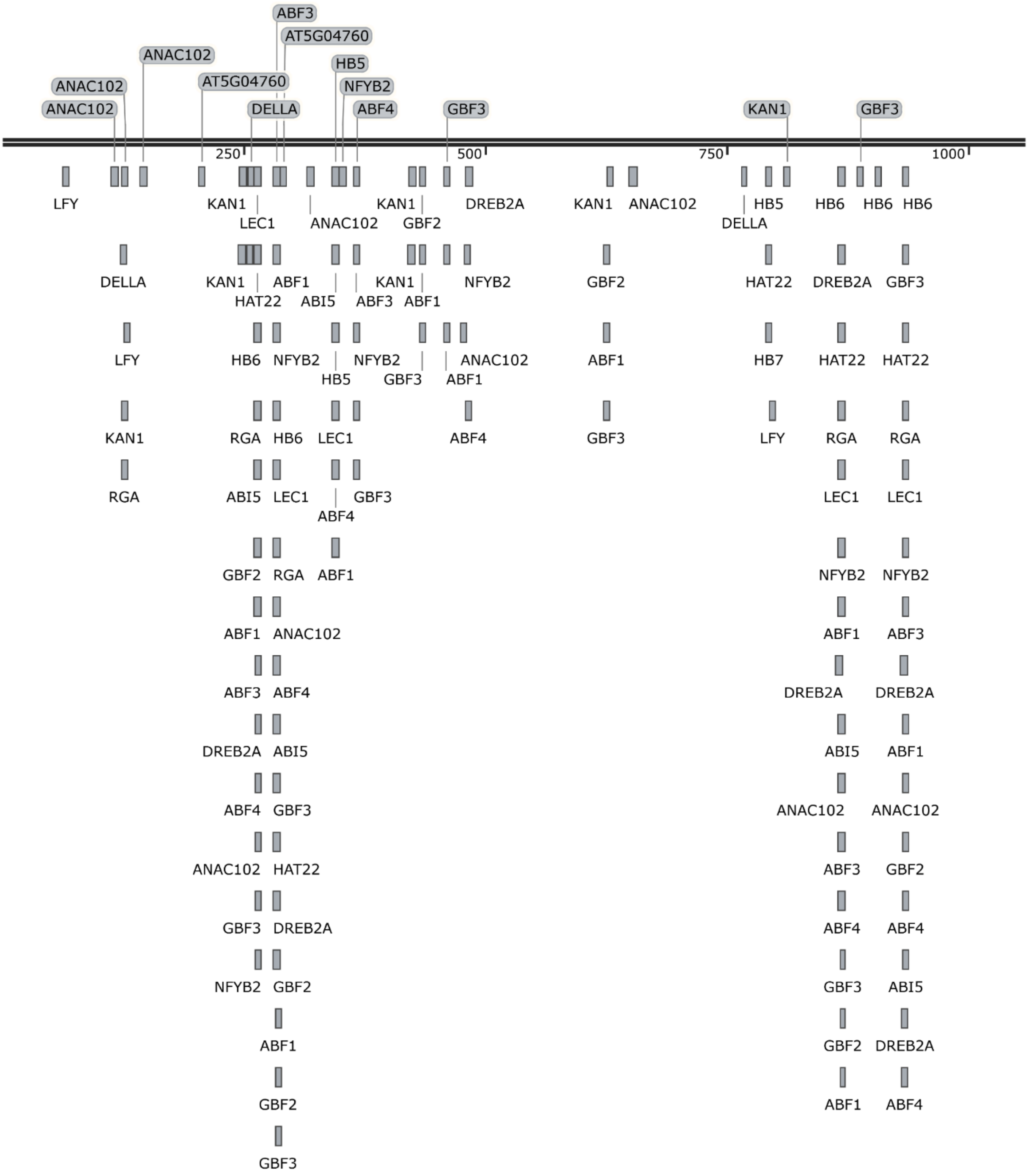

Figure S5. Mapping of the *PsABI5* gene promoter**.**
